# Supplementary material for: Trends and correlates of low HIV knowledge among ever-married women of reproductive age: Evidence from cross-sectional Bangladesh Demographic and Health Survey 1996–2014
Source: PLoS One. 2023 May 25;18(5):e0286184. doi: 10.1371/journal.pone.0286184 (PMC10212160; doi:10.1371/journal.pone.0286184)
Supplement: S1 File — (DOCX) [file pone.0286184.s001.docx]

S**1 Fig.** Trends of HIV knowledge among all women (both who ever heard and never heard of HIV) from 1996 to 2014

**S1 Table.** Knowledge of HIV among ever-married women from 1996 to 2014

| **Questions** | **1996**  **(n=1781)** | **1999**  **(n=3660)** | **2004**  **(n=7235)** | **2007**  **(n=7687)** | **2011**  **(n=12512)** | **2014**  **(n=12593)** |
| --- | --- | --- | --- | --- | --- | --- |
| People can reduce their chance of getting the AIDS virus by using a condom every time they have sex, yes % (n) | 5.5 (99) | 15.5 (601) | 36.5 (2707) | 47.4 (3764) | 63.3 (8011) | 59.7 (7475) |
| People can reduce their chance of getting the AIDS virus by having just one uninfected sex partner, who has no other sex partners, yes % (n) | 8.1 (150) | 6.1 (266) | 5.6 (426) | 48.2 (3709) | 73.4 (9262) | 73.2 (9117) |
| People get the AIDS virus from mosquito bites, no % | 98.7 (1758) | 99.5 (3642) | 81.3 (5966) | 39.8 (3252) | 46.8 (5920) | 54.1 (6821) |
| People get AIDS virus by unsafe blood transfusion, yes % | 3.5 (67) | 2.7 (105) | 11.5 (879) | 88.8 (6851) | 91.7 (11488) | 88.4 (11084) |
| People get the AIDS virus by using unsterilized needle or syringe, yes % (n) | 8.2 (158) | 9.1 (392) | 23.6 (1802) | 88.0 (6765) | 92.3 (11574) | 90.0 (11294) |
| It is possible a healthy-looking person can have AIDS virus, yes % (n) | 67.8 (1228) | 68.0 (2517) | 70.4 (5191) | 77.6 (6030) | 70.7 (8913) | 67.6 (8582) |
| There are ways to avoid AIDS, yes % (n) | 31.5 (580) | 54.7 (2090) | 75.3 (5548) | - | - | - |
| AIDS can avoid by abstaining from sex, yes % (n) | 5.6 (94) | 7.5 (291) | 11.1 (837) | 47.4 (3676) |  |  |
| AIDS can avoid by avoiding sex with prostitute, yes % (n) | 12.1 (231) | 17.6 (707) | 16.7 (1266) | - | - | - |
| AIDS can spread by kissing, no % (n) | 99.1 (1766) | 99.5 (3641) | 81.5 (5975) | - | - | - |
| People can get the AIDS virus because of witchcraft or other supernatural means, no % (n) | - | - | - | - | 78.1 (9849) | 76.3 (9565) |
| People get AIDS by sharing food with a person who has AIDS, no % (n) | - | - | - | 44.3 (3598) | 55.2 (6951) | 51.6 (6498) |
| AIDS transmitted from mother to her baby during pregnancy, yes % (n) | - | - | - | - | 84.9 (10679) | 79.8 (10087) |
| AIDS transmitted from mother to her baby during delivery, yes % (n) | - | - | - | - | 69.1 (8791) | 62.8 (7881) |
| AIDS transmitted from mother to her baby by breastfeeding, yes % (n) | - | - | - | - | 81.1 (10166) | 79.2 (9929) |
| People avoid AIDS by Traditional healer, no % (n) | 99.9 (1779) | 99.2 (3635) | 80.4 (5904) | - | - | - |
| AIDS is a fatal disease, yes % (n) | 60.6 (1096) | - | - | - | - | - |
| AIDS can avoid by avoid sex with homosexual, yes % (n) | - | 0.1 (27) | 0.5 (40) | - | - | - |
| AIDS can avoid by limiting sex with marriage or avoid sex with many partners, yes % (n) | - | 5.9 (230) | 17.8 (1333) | - | - | - |
| AIDS can avoid by avoiding sex with intravenous drug users, yes % (n) | - | 1.1 (48) | 1.2 (92) | - | - | - |

**S2 Table.** Correlates of low HIV knowledge among ever-married women from 1996 to 2014 (Simple logistic regression model)

| **Characteristics** | **1996**  **OR**  **(95 % CI)** | **1999**  **OR**  **(95 % CI)** | **2004**  **OR**  **(95 % CI)** | **2007**  **OR**  **(95 % CI)** | **2011**  **OR**  **(95 % CI)** | **2014**  **OR**  **(95 % CI)** | **Pooled data**  **OR**  **(95 % CI)** |
| --- | --- | --- | --- | --- | --- | --- | --- |
| **Age of respondent** | | | | | | |  |
| 15-19 years | Ref. | Ref. | Ref. | Ref. | Ref. | Ref. | Ref. |
| 20-29 years | 0.55**  (0.39, 0.78) | 0.54***  (0.44, 0.67) | 0.82**  (0.72, 0.94) | 0.96  (0.83, 1.12) | 0.84**  (0.74, 0.94) | 0.96  (0.85, 1.09) | 0.83***  (0.78, 0.88) |
| 30-39 years | 0.51***  (0.36, 0.72) | 0.58***  (0.46, 0.73) | 1.00  (0.86, 1.18) | 1.07  (0.92, 1.25) | 0.83**  (0.73, 0.94) | 0.99  (0.88, 1.12) | 0.88***  (0.82, 0.94) |
| 40-49 years | 0.59*  (0.39, 0.89) | 0.81  (0.63, 1.05) | 1.37**  (1.12, 1.68) | 1.27*  (1.06, 1.53) | 0.96  (0.83, 1.11) | 1.14  (0.99, 1.30) | 1.04  (0.96, 1.12) |
| **Age at first marriage** | | | | | | |  |
| < 15 years | Ref. | Ref. | Ref. | Ref. | Ref. | Ref. | Ref. |
| 15-17 years | 0.55***  (0.42, 0.40) | 0.76**  (0.65, 0.91) | 0.65***  (0.58, 0.73) | 0.78***  (0.71, 0.87) | 0.77***  (0.71, 0.84) | 0.83***  (0.76, 0.90) | 0.74***  (0.71, 0.77) |
| ≥ 18 years | 0.31***  (0.23, 0.41) | 0.38***  (0.31, 0.46) | 0.42***  (0.36, 0.49) | 0.43***  (0.38, 0.49) | 0.57***  (0.51, 0.63) | 0.62***  (0.56, 0.69) | 0.49***  (0.47, 0.52) |
| **Respondent’s education** | | | | | | |  |
| Illiterate | 9.59***  (6.25, 14.70) | 4.09***  (3.14, 5.31) | 4.18***  (3.59, 4.88) | 3.54***  (3.06, 4.10) | 2.46***  (2.19, 2.76) | 2.18***  (1.93, 2.46) | 3.07***  (2.88, 3.27) |
| Primary | 4.26***  (3.17, 5.73) | 3.24***  (2.63, 3.99) | 2.63***  (2.33, 2.97) | 2.41***  (2.14, 2.72) | 1.34***  (1.19, 1.52) | 1.92***  (1.76, 2.09) | 2.18***  (2.08, 2.89) |
| Secondary  or higher | Ref. | Ref. | Ref. | Ref. | Ref. | Ref. | Ref. |
| **Religion** | | | | | | |  |
| Others | Ref. | Ref. | Ref. | Ref. | Ref. | Ref. | Ref. |
| Muslim | 1.07  (0.77, 1.47) | 1.01  (0.79, 1.29) | 0.94  (0.76, 1.17) | 1.10  (0.87, 1.38) | 1.07  (0.92, 1.26) | 0.89  (0.76, 1.04) | 1.05  (0.96, 1.14) |
| **Employment status** | | | | | | |  |
| Unemployed | 0.87  (0.63, 1.10) | 1.24*  (1.03, 1.50) | 0.94  (0.84, 1.06) | 1.01  (0.91, 1.13) | 1.13*  (1.02, 1.26) | 0.96  (0.89, 1.05) | 0.98  (0.93, 1.02) |
| Employed | Ref. | Ref. | Ref. | Ref. | Ref. | Ref. | Ref. |
| **Current marital status** | | | | | | | |
| ^¶^Other | 1.30  (0.84, 2.01) | 1.99***  (1.43, 2.76) | 1.68***  (1.36, 2.08) | 1.54***  (1.24, 1.90) | 1.32***  (1.13, 1.54) | 1.26*  (1.05, 1.51) | 1.45***  (1.33, 1.59) |
| Married | Ref. | Ref. | Ref. | Ref. | Ref. | Ref. | Ref. |
| **Media exposure** | | | | | | |  |
| No | 5.43***  (3.38, 8.71) | 3.07***  (2.32, 4.06) | 5.06***  (4.02, 6.36) | 1.03  (0.93, 1.15) | 1.63***  (1.47, 1.80) | 1.83***  (1.66, 2.02) | 1.91***  (1.79, 2.04) |
| Yes | Ref. | Ref. | Ref. | Ref. | Ref. | Ref. | Ref. |
| **Use of condom during sexual intercourse** | | | | | | |  |
| No | 2.14***  (1.54, 2.98) | 2.84***  (2.32, 3.47) | 2.90***  (2.34, 3.59) | 3.11***  (2.55, 2.81) | 1.80***  (1.57, 2.07) | 1.77***  (1.54, 2.02) | 2.15***  (2.01, 2.32) |
| Yes | Ref. | Ref. | Ref. | Ref. | Ref. | Ref. | Ref. |
| **Sex of the household head** | | | | | | | |
| Male | Ref. | Ref. | Ref. | Ref. | Ref. | Ref. | Ref. |
| Female | 0.97  (0.67, 1.39) | 1.23  (0.95, 1.59) | 1.18  (0.98, 1.42 | 0.90  (0.76, 1.07) | 0.94  (0.84, 1.06) | 1.06  (0.94, 1.20) | 1.04  (0.98, 1.12) |
| **Type of place of residence** | | | | | | |  |
| Urban | Ref. | Ref. | Ref. | Ref. | Ref. | Ref. | Ref. |
| Rural | 2.40***  (1.78, 3.25) | 2.39***  (1.99, 2.85) | 2.14***  (1.84, 2.50) | 0.53***  (0.45, 0.61) | 1.45***  (1.31, 1.60) | 1.58***  (1.42, 1.76) | 1.70***  (1.61, 1.81) |
| **Wealth Quintile** | | | | | | |  |
| Richest | Ref. | Ref. | Ref. | Ref. | Ref. | Ref. | Ref. |
| Richer | 2.70***  (2.00, 3.64) | 2.36***  (1.94, 2.88) | 2.00***  (1.74, 2.31) | 1.73***  (1.48, 2.02) | 1.50***  (1.35, 1.67) | 1.40***  (1.26, 1.57) | 1.61***  (1.52, 1.71) |
| Middle | 5.27***  (3.15, 8.80) | 3.27***  (2.55, 4.21) | 3.18***  (2.68, 3.76) | 2.69***  (2.27, 3.19) | 1.80***  (1.60, 2.02) | 1.86***  (1.64, 2.10) | 2.11***  (1.97, 2.25) |
| Poorer | 6.90***  (4.10, 11.60) | 3.11***  (2.43, 3.97) | 4.42***  (3.62, 5.40) | 3.22***  (2.67, 3.88) | 2.31***  (2.03, 2.63) | 2.15***  (1.87, 2.47) | 2.60***  (2.41, 2.80) |
| Poorest | 7.23***  (3.51, 14.90) | 4.98***  (3.44, 7.19) | 5.99***  (4.68, 7.66) | 3.77***  (3.00, 4.73) | 2.47***  (2.14, 2.84) | 2.27***  (1.95, 2.64) | 2.90***  (2.66, 3.16) |
| **Administrative division** | | | | | | |  |
| Barisal | Ref. | Ref. | Ref. | Ref. | Ref. | Ref. | Ref. |
| Dhaka | 1.10  (0.59, 2.10) | 1.06  (0.73, 1.53) | 0.72*  (0.53, 0.98) | 0.80  (0.60, 1.07) | 1.28*  (1.05, 1.57) | 0.75**  (0.63, 0.90) | 0.95  (0.85, 1.06) |
| Chattogram | 1.16  (0.62, 2.18) | 1.37  (0.84, 2.21) | 0.94  (0.69, 1.27) | 1.18  (0.89, 1.57) | 1.35**  (1.09, 1.67) | 0.82  (0.68, 1.00) | 1.09**  (0.97, 1.23) |
| Khulna | 1.15  (0.56, 2.36) | 1.13  (0.76, 1.67) | 0.62**  (0.45, 0.85) | 0.77  (0.56, 1.06) | 1.37**  (1.13, 1.66) | 0.74**  (0.61, 0.92) | 0.91  (0.80, 1.02) |
| Rajshahi | 1.17  (0.57, 2.40) | 1.43  (0.97, 2.10) | 0.93  (0.69, 1.25) | 0.80  (0.59, 1.08) | 1.41**  (1.15, 1.74) | 0.75**  (0.61, 0.92) | 1.03  (0.91, 1.16) |
| Rangpur | - | - | - | - | 1.28*  (1.04, 1.58) | 0.81*  (0.66, 0.99) | 1.02  (0.88, 1.20) |
| Sylhet | 1.87  (0.89, 3.95) | 1.09  (0.67, 1.48) | 1.00  (0.51, 1.04) | 1.32  (0.98, 1.78) | 1.44**  (1.16, 1.79) | 1.07  (0.85, 1.34) | 1.05*  (0.93, 1.20) |
| **Survey year** | - | - | - | - | - | - |  |
| 1996 | - | - | - | - | - | - | Ref. |
| 1999 | - | - | - | - | - | - | 0.71***  (0.59, 0.86) |
| 2004 | - | - | - | - | - | - | 0.76**  (0.64, 0.91) |
| 2007 | - | - | - | - | - | - | 0.65***  (0.54, 0.77) |
| 2011 | - | - | - | - | - | - | 0.45***  (0.38, 0.54) |
| 2014 | - | - | - | - | - | - | 0.57***  (0.49, 0.68) |

^¶^Widowed /Divorced/Not living together, *p<0.05, **p<0.01, ***p<0.001, OR=Odds Ratio, CI=Confidence Interval

**S3 Table.** Correlates of “low” HIV knowledge among ever-married women from 1996 to 2014 (Multiple logistic regression model)

| **Characteristics** | **1996**  **AOR**  **(95% CI)** | **1999**  **AOR**  **(95% CI)** | **2004**  **AOR**  **(95% CI)** | **2007**  **AOR**  **(95% CI)** | **2011**  **AOR**  **(95% CI)** | **2014**  **AOR**  **(95% CI)** | **Pooled data**  **AOR**  **(95% CI)** |
| --- | --- | --- | --- | --- | --- | --- | --- |
| **Age categories** |  |  |  |  |  |  |  |
| 15-19 years | Ref. | Ref. | Ref. | Ref. | Ref. | Ref. | Ref. |
| 20-29 years | 0.62*  (0.43, 0.90) | 0.60***  (0.48, 0.75) | 0.88  (0.76, 1.01) | 0.99  (0.85, 1.15) | 0.81***  (0.72, 0.9) | 0.88*  (0.78, 0.98) | 0.82***  (0.78, 0.88) |
| 30-39 years | 0.52***  (0.37, 0.74) | 0.69**  (0.55, 0.87) | 1.05  (0.88, 1.24) | 1.08  (0.93, 1.27) | 0.76***  (0.67, 0.87) | 0.86*  (0.76, 0.97) | 0.82***  (0.77, 0.88) |
| 40-49 years | 0.46***  (0.30, 0.71) | 0.86  (0.64, 1.15) | 1.39**  (1.15, 1.69) | 1.32**  (1.09, 1.60) | 0.89  (0.78, 1.03) | 0.96  (0.84, 1.11) | 0.96  (0.89, 1.03) |
| **Age at first marriage** | | | | | | |  |
| <15 years | Ref. | Ref. | Ref. | Ref. | Ref. | Ref. | Ref. |
| 15-17 years | 0.74*  (0.57, 0.97) | 0.96  (0.80, 1.16) | 0.82**  (0.73, 0.92) | 0.98  (0.88, 1.09) | 0.88**  (0.81, 0.96) | 0.88**  (0.81, 0.96) | 0.87***  (0.83, 0.90) |
| ≥18 years | 0.54***  (0.41, 0.70) | 0.70**  (0.57, 0.86) | 0.69***  (0.60, 0.81) | 0.75***  (0.65, 0.86) | 0.76***  (0.69, 0.84) | 0.75***  (0.68, 0.83) | 0.71***  (0.67, 0.75) |
| **Education level** |  |  |  |  |  |  |  |
| Illiterate | 15.21***  (9.75, 23.72) | 6.27***  (4.89, 8.04) | 3.68***  (3.12, 4.34) | 4.35***  (3.67, 5.15) | 3.75***  (3.32, 4.25) | 3.66***  (3.21, 4.16) | 4.30***  (4.01, 4.61) |
| Primary | 4.84***  (3.59, 6.52) | 3.35***  (2.76, 4.07) | 2.04***  (1.78, 2.33) | 2.35***  (2.08, 2.65) | 2.04***  (1.87, 2.23) | 2.14***  (1.95, 2.35) | 2.28***  (2.17, 2.40) |
| Secondary  or higher | Ref. | Ref. | Ref. | Ref. | Ref. | Ref. | Ref. |
| **Employment** |  |  |  |  |  |  |  |
| Unemployed |  | 1.59***  (1.32, 1.92) | - | - | 1.22***  (1.1, 1.37) | - | - |
| Employed | - | Ref. | - | - | Ref. | - | - |
| **Current marital status** | | | | | | | |
| Married | - | Ref. | Ref. | Ref. | Ref. | Ref. | Ref. |
| ^¶^Other | - | 1.88***  (1.34, 2.65) | 1.21  (0.95, 1.55) | 1.24  (0.98, 1.54) | 1.23**  (1.06, 1.44) | 1.13  (0.94, 1.34) | 1.22***  (1.11, 1.33) |
| **Media exposure** |  |  |  |  |  |  |  |
| No | 5.31***  (3.32, 8.50) | 4.06***  (3.14, 5.24) | 5.03***  (4.00, 6.31) | - | 1.64***  (1.48, 1.81) | - | 1.72***  (1.63, 1.88) |
|  | Ref. | Ref. | Ref. | - | Ref. | - | Ref. |
| **Use of condom** |  |  |  |  |  |  |  |
| No | 1.50*  (1.10, 2.06) | 1.90***  (1.51, 2.38) | 1.69***  (1.34, 2.13) | 1.98***  (1.61, 2.43) | 1.42***  (1.24, 1.64) | 1.29***  (1.13, 1.48) | 1.52***  (1.40, 1.64) |
| Yes | Ref. | Ref. | Ref. | Ref. | Ref. | Ref. | Ref. |
| **Type of place of residence** | | | | | | |  |
| Urban | Ref. | Ref. | Ref. | Ref. | Ref. | Ref. | Ref. |
| Rural | 2.30***  (1.75, 3.03) | 2.56***  (2.08, 3.16) | 1.72***  (1.45, 2.03) | 1.66***  (1.42, 1.95) | 1.17**  (1.05, 1.3) | 1.43***  (1.28, 1.6) | 1.53***  (1.43, 1.62) |
| **Wealth Quintile** |  |  |  |  |  |  |  |
| Richest | Ref. | Ref. | Ref. | Ref. | Ref. | Ref. | Ref. |
| Richer | 2.46***  (1.83, 3.31) | 1.83***  (1.47, 2.26) | 1.49***  (1.28, 1.74) | 1.22**  (1.05, 1.41) | 1.35***  (1.21, 1.51) | 1.24***  (1.1, 1.4) | 1.44***  (1.36, 1.53) |
| Middle | 4.28***  (2.56, 7.17) | 2.40***  (1.81, 3.18) | 2.26***  (1.89, 2.71) | 1.94***  (1.63, 2.32) | 1.56***  (1.38, 1.77) | 1.64*** (1.44, 1.86) | 1.82***  (1.70, 1.95) |
| Poorer | 6.28***  (3.61, 10.93) | 2.29***  (1.77, 2.97) | 3.15***  (2.54, 3.91) | 2.48***  (2.04, 3.03) | 2.15***  (1.87, 2.47) | 2.05*** (1.77, 2.38) | 2.22***  (2.06, 2.41) |
| Poorest | 4.58***  (2.19, 9.55) | 3.61***  (2.48, 5.25) | 4.21***  (3.29, 5.39) | 3.13***  (2.47, 3.97) | 2.3***  (1.96, 2.71) | 2.51*** (2.11, 2.97) | 2.61***  (2.38, 2.85) |
| **Administrative division** | | | | | | |  |
| Barisal |  |  | Ref. |  | Ref. | Ref. | Ref. |
| Dhaka |  |  | 0.75*  (0.57, 0.98) |  | 1.36**  (1.13, 1.63) | 1.16  (0.96, 1.4) | 1.03  (0.93, 1.15) |
| Chattogram |  |  | 1.17  (0.89, 1.54) |  | 1.65***  (1.35, 2.02) | 1.23*  (1.03, 1.48) | 1.40***  (1.27, 1.54) |
| Khulna |  |  | 0.55***  (0.41, 0.73) |  | 1.25*  (1.03, 1.52) | 0.83*  (0.69, 0.98) | 0.87**  (0.79, 0.96) |
| Rajshahi |  |  | 0.96  (0.72, 1.29) |  | 1.64***  (1.35, 2) | 1.05  (0.87, 1.27) | 1.21***  (1.10, 1.34) |
| Rangpur |  |  | - |  | 1.82***  (1.48, 2.22) | 1.48**  (1.18, 1.85) | 1.50***  (1.31, 1.74) |
| Sylhet |  |  | 1.23  (0.90, 1.76) |  | 1.48***  (1.22, 1.80) | 1.06  (0.87, 1.29) | 1.26***  (1.13, 1.40) |
| **Survey year** |  |  |  |  |  |  |  |
| 1996 |  |  |  |  |  |  | Ref. |
| 1999 | - | - | - | - | - | - | 0.48***  (0.41, 0.57) |
| 2004 | - | - | - | - | - | - | 0.26***  (0.23, 0.31) |
| 2007 | - | - | - | - | - | - | 0.19***  (0.16, 0.22) |
| 2011 | - | - | - | - | - | - | 0.16***  (0.13, 0.18) |
| 2014 | - | - | - | - | - | - | 0.20***  (0.17, 0.23) |
| Hosmer-Lemeshow  p-value | 0.6605 | 0.7858 | 0.1166 | 0.8464 | 0.1044 | 0.0048 | <0.001 |
| AUC | 0.9345 | 0.8886 | 0.8352 | 0.7760 | 0.7452 | 0.7258 | 0.8102 |

¶Widowed /Divorced/Not living together, *p<0.05, **p<0.01, ***p<0.001, AOR=Adjusted Odds Ratio, CI=Confidence Interval
