# Supplementary material for: Trends and correlates of low HIV knowledge among ever-married women of reproductive age: Evidence from cross-sectional Bangladesh Demographic and Health Survey 1996–2014
Source: PLoS One. 2023 May 25;18(5):e0286184. doi: 10.1371/journal.pone.0286184 (PMC10212160; doi:10.1371/journal.pone.0286184)
Supplement: Attachment — Submitted filename: Response to Reviewers.docx [file pone.0286184.s002.docx]

Reviewers' comments:

Reviewer #1: Dear Authors,

Thanks for choosing an interesting topic for the manuscript “Trends and correlates of low HIV knowledge among ever-married women of reproductive age: Evidence from cross-sectional Bangladesh Demographic and Health Survey 1996-2014”. I have reviewed your article and have few concerns to improve methodological rigor of your research

Abstract

Line # 47: Within the results, kindly also mention AOR for the year 1996
I would suggest to rephrase the conclusion from lines # 49-51, e.g. instead of ‘reduce’, reduction of disparities between urban-rural and poorest-richest quintiles may address the low level of HIV knowledge …

Response: We included 1996 as the reference category (AOR =1) and compared it with other categories. As per your suggestion, we have rephrased the conclusion section as-

*“The prevention of early marriage, the inclusion of HIV-related topics in the curriculum, reduction of disparities between urban-rural and poorest-richest quintiles may address the “low” level of HIV knowledge among ever-married Bangladeshi women.”* *(page 2, Lines: 48-50).*

Introduction

Line # 69: Can you please explain “Rohingya” for understanding of general readers?

Response: Explained in parenthesis as follows-

*“an ethnic group who mostly* *follow Islam and resided in Rakhine State, Myanmar” (page 3, Lines: 68-69).*

I think authors should highlight the prevailing misconceptions and rumors associated with lack of knowledge about HIV.

Response: We highlighted the prevailing misconceptions and rumors regarding HIV among ever-married women as follows-

*“In addition, many misconceptions and rumors regarding HIV including people get HIV from mosquito bite, sharing foods with a person who has AIDS and by witchcraft or supernatural means are exist among ever-married women” (pages 3-4, Lines: 83-85).*

Line # 93-94: I disagree with authors regarding the given explanation to not considering the ‘ever-heard of HIV’. In DHS, this is an opening question, which applies to only those women, who had ever heard of HIV and excluding those who had not heard HIV. Subsequently, questions related to HIV knowledge are prompted. Authors’ justification is quite vague. I would recommend to highlight the gaps in existing literature, which are being bridged due to this research. Most importantly, how this study is difference from the earlier study (Sheikh, 2017), what value your research is adding into literature? Authors should answer these important questions.

Response: Thanks for your concerns. We did not exclude ever-heard of HIV. We analyzed the data for this study based on the sample of ever-married women who ever-heard of HIV. We agree with you that ever-heard of HIV is an opening question in BDHS and the women who responded “yes” subsequently asked the knowledge-related questions (Table 1).

In the cited paper, they mentioned the knowledge if the women ever-heard of HIV and otherwise no knowledge. But in our study, we calculated the knowledge score based on the individual knowledge question response, which we think makes our study differs from the cited study.

According to your comment, we also included the women of never-heard of HIV and did the analysis as a sensitivity analysis. We included the results of sensitivity analysis and presented the trends figure and correlates table as supplementary materials.

Methodology
Line # 103-104: Here you have taken sample of ever-married women of reproductive age (15-49 years) who ever-heard of HIV. Isn’t it contradictory with your stated research objectives.

Response: We mentioned our study objective before the methodology section in the revised version.

*“We included ever-married women of reproductive age as our main analysis but in the revised version we included both samples as sensitivity analysis.” (Page 4, Lines: 97-99)*

Line # 105: Therefore, our sample sizes for this study were 1781, 3660, 7235, 7687 2011, 12512, and 12593 in the survey years … here ‘2011’ seems typo error and must be omitted.

Response: Thanks. We omitted the typo in the revised version.

Sampling design: Was that design with 600 clusters (207 urban and 393 rural) remained same throughout varied BDHSs?

Response: The number of clusters throughout the BDHSs are not the same across time. The variations in the number of clusters are largely based on the population census used for drawing samples. As the population census was conducted over roughly 10 years interval in Bangladesh, the selected clusters are likely to change after this period. In addition, the number of clusters may change between the successive round of BDHSs as well depending on the sampling strategy and/or selection criteria.

We have revised as—

*“The sampling frame was a complete list of enumeration areas (EAs) or clusters, consisting of either a village, part of a village, or a group of villages. In the first stage of sampling, in 1996, 313 clusters (71 urban and 242 rural), in 1996, 341 clusters (99 urban and 242 rural), in 2004, 361 clusters (122 urban and 239 rural), in 2007, 364 clusters (136 urban and 228 rural), in 2011, 600 clusters (207 urban and 393 rural) and in 2014, 619 clusters (226 urban and 393 rural) were selected throughout Bangladesh using probability proportional to size (PPS) sampling.” (Page) (Page 5, Lines: 123-128).*

Outcome measure:
Authors are advised to highlight why the series of questions for HIV related knowledge is not unform across the varied BDHSs?

Response: We acknowledge that the knowledge questions are not uniform across all BDHSs. This is expected as the BDHS questionnaire is upgradable depending on the new knowledge, indicators, aspects and issues generated/identified from up-to-date research findings. We have discussed this in the discussion section. The added sentences are as follows:

*“All the HIV-related knowledge questions were not uniform across the surveys because the BDHS questionnaire is upgradable depending on the new knowledge, indicators, aspects, and issues generated/identified from up-to-date research findings.” (Page 5, Lines: 140-142)*

Statistical analysis

Kindly mention which statistical software was used for analysis.

Response: We have mentioned the name of the software in the revised version. *(Page 8, Lines: 198-199).*

I am interested to explore how pooled data was constructed and analyzed

Response: Thank you so much for your concern. The pooled data were constructed by combining all the individual survey data i. e by appending all six surveys datasets into a single dataset.
Lastly, I would recommend to add future research directions and implications of this research

Response: Thanks for your recommendations. We have added the implications and future research directions in the revised version as follows:

*“This study would be the directive for initiating long-term interventions for improving the level of HIV-related knowledge considering the identified correlates. Future research is warranted to establish the causal relationship of level of HIV knowledge and individual, community and sociodemographic factors.” (Page 17, Lines: 374-378).*
